# Racial differences as predictors of outcomes in young patients with multiple myeloma

**DOI:** 10.1038/s41408-022-00708-3

**Published:** 2022-07-27

**Authors:** Alicia Bao, Qiuhong Zhao, Elizabeth Merritt, Naresh Bumma, Srinivas Devarakonda, Abdullah M. Khan, Elvira Umyarova, Ashley E. Rosko, Don M. Benson, Francesca Cottini

**Affiliations:** 1 College of Medicine, The Ohio State University, Columbus, OH USA; 2 Department of Internal Medicine, Division of Hematology, College of Medicine, The Ohio State University, Columbus, OH USA; 3 The Ohio State University, Columbus, OH USA

**Keywords:** Disease-free survival, Epidemiology

## Introduction

Dear editors, Dong et al. [1] recently showed in the SEER-Medicare database that non-Hispanic Black patients (NHBP) with multiple myeloma (MM) older than 65 years have better survival outcomes than age-matched non-Hispanic White patients (NHWP) when receiving similar treatments. Despite a median age of diagnosis of 69 years, 10% of patients develop MM before age 50. These younger patients usually present with favorable disease status and can tolerate more intensive treatment, leading to a longer survival than their older counterparts [2, 3]. A few studies have examined outcomes and disease characteristics in the younger MM patient population [3, 4], with evidence that young NHWP and NHBP generally outlive older patients within their racial group [5], but none have evaluated survival differences between races. Our study fills this gap by specifically investigating the impact of race and cytogenetic abnormalities in patients with MM diagnosed before age 50.

## Methods

258 individuals under 50 years of age diagnosed with MM between 1992 and 2019 at The Ohio State University Comprehensive Cancer Center, in Columbus, Ohio were included in a retrospective single-center study, approved by The Ohio State University Institutional Review Board (2019C0091). Data pertaining to patient demographics and MM characteristics were collected in a coded database. High-risk chromosomal abnormalities (HRCA) were defined as the presence of 1q21+, t(4;14), t(14;16), t(14;20), or del(17p). Descriptive statistics were used to summarize patient and disease characteristics; chi-square or Fisher’s exact, and Wilcoxon rank-sum tests were used to compare distributions of characteristics between NHBP and NHWP. The endpoints of the studies were overall survival (OS) and progression-free survival (PFS). OS was calculated from the date of diagnosis or first transplant to the date of death, censoring those alive at last known follow-up. PFS was calculated from the date of diagnosis or first transplant to date of disease progression or death (whichever occurred first), censoring those without progression at the date of last clinical assessment. OS/PFS rates were estimated using Kaplan–Meier method. Log-rank tests were used to test survival functions, while Cox proportional models to evaluate the association between patient characteristics and OS/PFS. Stata 16 was used for all the analyses and all the tests were 2-sided with type I error at 0.05.

## Results

In our institutional database, we identified 258 individuals who developed MM before age 50 (median age = 46; range: 17–50 years) who were either NHBP (60/258, 23.3%) or NHWP (198/258, 76.7%). Patient and disease characteristics are summarized in Supplementary Table S1. The median length of follow-up was 7.8 years. In all, median OS was 9.4 years (95% CI: 7.6–12.5 years), which is longer than the reported OS in older MM patients [2, 3]. Most of the patients were male (131/198, 66.2% of NHWP and 34/60, 56.7% of NHBP), presented with International Staging System (ISS) stage I disease (66/198, 40.0% of NHWP and 23/60, 48.9% of NHBP), and had IgG disease (89/198, 44.9% of NHWP and 32/60, 53.3% of NHBP). 23/198 NHWP (11.6%) and 2/60 NHBP (3.3%) were diagnosed with MM between 1992 and 2002, while the remaining 233 patients (175/198, 88.4% of NHWP and 58/60, 96.7% of NHBP) were diagnosed after January 2003 (*P* = 0.08). MM was the most common presentation, with 45/198 NHWP (22.7%) and 10/60 NHBP (16.7%) presenting with extramedullary myeloma (EMM) or primary plasma cell leukemia. Of the 210 patients with fluorescence in situ hybridization (FISH) studies at diagnosis, most of the patients (111/159, 68.9% of NHWP and 36/51, 73.5% of NHBP) had no HRCA, 49 (37/159, 23.0% of NHWP and 12/51, 24.5% of NHBP) had one HRCA, and 14 (13/159, 8.1% of NHWP and 1/51, 2.0% of NHBP) had more than 2 HRCAs. NHWP had a statistically significant predominance of del(13q) compared with NHBP (62/159, 39.0% of NHWP and 10/51, 19.6% of NHBP; *P* = 0.01). Of 221 patients with available family history, 101/172 NHWP (58.7%) and 21/49 NHBP (42.9%) had at least one family member affected by any type of cancer (*P* = 0.05), including 6/172 NHWP (3.5%) and 1/49 NHBP (2.0%) with family history of MM or precursor forms (*P* = 0.99). Moreover, 22/176 NHWP (12.5%) and 1/50 NHBP (2.0%) had a second malignancy (Supplementary Table S2), either before or after MM diagnosis (*P* = 0.03), with 4/22 cases of non-melanoma skin cancers in NHWP. This difference was not retained after stratifying by time of occurrence (before MM diagnosis, *P* = 0.70; after MM diagnosis, *P* = 0.08). Finally, 79/193 NHWP (40.9%) and 28/58 NHBP (48.2%) were either active or former smokers at time of MM diagnosis (*P* = 0.37).

Most of the patients (115/198, 58.1% of NHWP and 37/60, 61.7% of NHBP) received induction therapy with bortezomib (V), lenalidomide (R), and dexamethasone (D) either as double or triple therapy (VD-RD-VRD), while 83/198 NHWP (41.9%) and 23/60 NHBP (38.3%) received other induction regimens, including chemotherapy or other combinations. In our cohort, NHWP and NHBP underwent transplant at similar rates (185/198, 93.4% of NHWP and 53/60, 88.3% of NHBP; *P* = 0.11) and maintenance therapy was started in 95/185 NHWP (51.4%) and 21/53 NHBP (43.4%; *P* = 0.31).

**Fig. 1:**
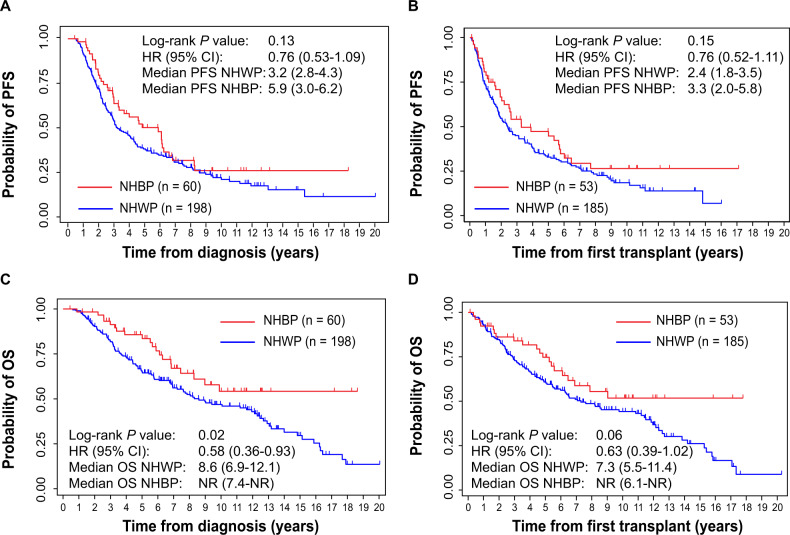
Outcomes of non-Hispanic White patients (NHWP) and non-Hispanic Black patients (NHBP) with MM diagnosed before age 50. Top panel: Kaplan–Meier curves of progression-free survival (PFS) from diagnosis in *n* = 258 patients (**A**) or from first transplant in *n* = 238 patients (**B**). Bottom panel: Kaplan–Meier curves of overall survival (OS) from diagnosis in *n* = 258 patients (**C**) or from first transplant in *n* = 238 patients (**D**). *P* log-rank p-value, HR Hazard Ratio, CI Confidence Interval, NR not reached.

Median PFS from diagnosis was 3.2 years (95% CI: 2.8–4.3) in NHWP versus 5.9 years (95% CI: 3.0–6.2) in NHBP (HR = 0.76, 95% CI: 0.53–1.09, *P* = 0.13), as shown in Fig. 1A and Supplementary Table S3. Median PFS from first transplant was 2.4 years (95% CI: 1.8–3.5) in NHWP versus 3.3 years (95% CI: 2.0–5.8) in NHBP (HR = 0.76, 95% CI: 0.52–1.11, *P* = 0.15), as shown in Fig. 1B and Supplementary Table S3. The impact of race on PFS became statistically significant in patients who received VD-RD-VRD as induction regimen (Supplementary Fig. S1A, B), with median PFS from diagnosis of 3.4 years (95% CI: 2.6–4.4) in NHWP versus 6.2 years (95% CI: 4.0-not reached (NR)) in NHBP (HR = 0.59, 95% CI: 0.36–0.96, *P* = 0.03) and median PFS from first transplant of 2.7 years in NHWP (95% CI: 1.8–3.5) versus 6.0 years (95% CI: 3.3-NR) in NHBP (HR = 0.55, 95% CI: 0.32–0.95, *P* = 0.03). PFS from diagnosis or from first transplant were not significantly different between NHBP and NHWP who received other therapies as induction regimen (Supplementary Fig. S1C, D).

We then observed an inferior median OS from diagnosis of 8.6 years (95% CI: 6.9–12.1) in NHWP, while OS was NR (95% CI: 7.4-NR) in NHBP (HR = 0.58, 95% CI: 0.36–0.93, *P* = 0.02), as shown in Fig. 1C. 5-year OS in NHBP was 86% (95% CI: 73–93%) compared with 66% (95% CI: 58–72%) in NHWP (Supplementary Table S4). NHWP also had an inferior median OS from first transplant of 7.3 years (95% CI: 5.5–11.4) versus NR (95% CI: 6.1-NR) in NHBP (HR = 0.63, 95% CI: 0.39–1.02, *P* = 0.06), as shown in Fig. 1D and Supplementary Table S4. Similarly, in the subgroup of patients who received VD-RD-VRD induction, OS from diagnosis (HR = 0.46, 95% CI: 0.23–0.93, *P* = 0.03) or first transplant (HR = 0.53, 95% CI: 0.26–1.08, *P* = 0.08) were inferior in NHWP compared with NHBP (Supplementary Fig. S1E, F), while no difference in OS was noted using other induction regimens (Supplementary Fig. S1G, H). 5/198 NHWP (2.5%) and 0/60 NHBP (0%) died of complications of their second malignancy. Excluding all 23 patients (22 NHWP and 1 NHBP) with a second malignancy (Supplementary Fig. S2), OS from diagnosis remained inferior in NHWP compared with NHBP (HR = 0.60, 95% CI: 0.37–0.97, *P* = 0.03), suggesting that the second malignancy did not affect the outcomes of our studies. Other variables associated with OS were similar to patients older than 50 years [6], such as ISS stage III and the presence of t(4;14), 1q21+, and del(17p).

The presence of del(13q) was prognostically negative towards OS in all (*n* = 159) NHWP (HR = 2.74, 95% CI: 1.77–4.24, *P* < 0.001, Supplementary Fig. S3A), in NHWP (*n* = 99) treated with VD-RD-VRD regimen (HR = 2.70, 95% CI: 1.49–4.89, *P* = 0.001, Supplementary Fig. S3C) and in NHWP (*n* = 60) treated with other induction therapies (HR = 2.87, 95% CI: 1.50–5.51, *P* = 0.002, Supplementary Fig. S3E). The prognostic impact of del(13q) was instead borderline or not statistically significant towards OS in all (*n* = 51) NHBP (HR = 2.96, 95% CI: 0.91–9.63, *P* = 0.06, Supplementary Fig. S3B), in NHBP (*n* = 34) treated with VD-RD-VRD induction (HR = 4.64, 95% CI: 0.85–25.48, *P* = 0.07, Supplementary Fig. S3D), and in NHBP (*n* = 17) treated with other induction therapies (HR = 1.72, 95% CI: 0.36–10.89, *P* = 0.43, Supplementary Fig. S3F). In the multivariable analysis, after controlling for ISS stage III, HRCA, del(13q), and type of induction, the impact of race on OS was no longer significant (HR = 0.79, 95% CI: 0.42–1.49), suggesting that the favorable OS outcome seen in young NHBP can be due to a reduced incidence of high-risk features.

## Discussion

To our knowledge, this is the first study in the era of novel therapies to specifically evaluate the impact of race on outcomes for patients with MM before age 50. Despite being a single-center study, our patient and disease characteristics are representative of the typical MM population, with NHB individuals comprising 23% of the study.

We observed that NHBP younger than 50 had statistically longer OS from diagnosis compared with NHWP, in agreement with recent studies focused on patients with MM over the age of 65 [1, 7]. Earlier studies reported inferior or similar survival outcomes [8–11] in the NHBP population, but mainly included older patients treated before the introduction of novel therapies [8], or with reduced access to standard-of-care induction regimens or frontline autologous stem cell transplant [9–11]. In our cohort, NHBP and NHWP underwent comparable induction therapy regimens and received transplant and maintenance therapy at similar rates. In the subgroup analysis based on induction regimen, we observed that VD-RD-VRD regimens were especially beneficial in terms of PFS and OS in NHBP compared with NHWP, while race did not impact PFS or OS in patients treated with alternative regimens.

We identified fewer HRCA features in NHBP, like prior studies [12]. Del(13q) was more common in NHWP and was negatively prognostic, independent of induction regimen, towards OS in NHWP, but not in NHBP. This suggests a more indolent course for young NHBP or possibly racial differences in the prognostic role of del(13q) in young patients with MM, contributing to better PFS and OS with the use of VD-RD-VRD therapies.

In our cohort, NHWP had a greater incidence of all second malignancies and a trend toward more second primary malignancies (SPM) compared with NHBP. The etiology of SPM in MM is likely multifactorial due to host, disease, and treatment-related factors [13]. Small studies revealed that the specific types of SPM often vary by race and NHWP tend to have more SPM than NHBP [14, 15]. Except for one patient, all the patients with SPM in our study underwent at least one transplant, with 5/13 cases of hematological second malignancies developing after anti-MM treatment. Therefore, the presence of SPM in young MM patients should be further evaluated in larger cohorts, given the hypothetical concern of genetic predisposition or anti-MM therapies as triggers for SPM in young patients with MM.

This study has some limitations. Firstly, only individuals who consented to be part of our MM registry were included in the study, therefore subjecting our study to selection bias. Race was also self-reported by the patients. Secondly, this was a retrospective analysis conducted at a single institution. The global incidence of MM in patients younger than age 50 is low; thus, to sufficiently power the study, patients treated over a broad time span (1992–2019) were included. The paradigm of MM treatments drastically evolved during this interval and almost 40% of our entire cohort did not receive VD-RD-VRD as their induction strategy. In those patients, race did not impact survival outcomes, underlining the importance of utilizing modern treatment regimens in all patients. Additionally, this study was not designed to investigate differences in social determinants of health or access to care; however, those variables might contribute to our results on MM survival.

In summary, NHBP diagnosed with MM before age 50 have longer PFS and OS than age-matched NHWP, when treated with similar therapies and VD-RD-VRD regimens. These findings, together with the reduced prevalence of high-risk features at diagnosis, underline the importance of providing standard-of-care therapies to NHBP to improve long-term outcomes.

## Supplementary information

Supplemental Material (tables and figures)

## Data Availability

All data generated or analyzed during this study are included in this published article [and its supplementary information files]. Supplementary information is available at Blood Cancer Journal’s website.

## References

[1] Dong J, Garacci Z, Buradagunta CS, D’Souza A, Mohan M, Cunningham A (2022). Black patients with multiple myeloma have better survival than white patients when treated equally: a matched cohort study. Blood Cancer J.

[2] Lenhoff S, Hjorth M, Westin J, Brinch L, Backstrom B, Carlson K (2006). Impact of age on survival after intensive therapy for multiple myeloma: a population-based study by the Nordic Myeloma Study Group. Br J Haematol.

[3] Ludwig H, Durie BG, Bolejack V, Turesson I, Kyle RA, Blade J (2008). Myeloma in patients younger than age 50 years presents with more favorable features and shows better survival: an analysis of 10 549 patients from the International Myeloma Working Group. Blood.

[4] Jurczyszyn A, Davila J, Kortum KM, Jayabalan DS, Vij R, Fiala M (2019). Multiple myeloma in patients up to 30 years of age: a multicenter retrospective study of 52 cases. Leuk Lymphoma.

[5] Ailawadhi S, Azzouqa AG, Hodge D, Cochuyt J, Jani P, Ahmed S (2019). Survival trends in young patients with multiple myeloma: a focus on racial-ethnic minorities. Clin Lymphoma Myeloma Leuk.

[6] Abdallah N, Rajkumar SV, Greipp P, Kapoor P, Gertz MA, Dispenzieri A (2020). Cytogenetic abnormalities in multiple myeloma: association with disease characteristics and treatment response. Blood Cancer J.

[7] Fillmore NR, Yellapragada SV, Ifeorah C, Mehta A, Cirstea D, White PS (2019). With equal access, African American patients have superior survival compared to white patients with multiple myeloma: a VA study. Blood.

[8] Waxman AJ, Mink PJ, Devesa SS, Anderson WF, Weiss BM, Kristinsson SY (2010). Racial disparities in incidence and outcome in multiple myeloma: a population-based study. Blood.

[9] Joshua TV, Rizzo JD, Zhang MJ, Hari PN, Kurian S, Pasquini M (2010). Access to hematopoietic stem cell transplantation: effect of race and sex. Cancer.

[10] Derman BA, Jasielec J, Langerman SS, Zhang W, Jakubowiak AJ, Chiu BC (2020). Racial differences in treatment and outcomes in multiple myeloma: a multiple myeloma research foundation analysis. Blood Cancer J.

[11] Bhatnagar V, Wu Y, Goloubeva OG, Ruehle KT, Milliron TE, Harris CG (2015). Disparities in black and white patients with multiple myeloma referred for autologous hematopoietic transplantation: a single center study. Cancer.

[12] Greenberg AJ, Philip S, Paner A, Velinova S, Badros A, Catchatourian R (2015). Racial differences in primary cytogenetic abnormalities in multiple myeloma: a multi-center study. Blood Cancer J.

[13] Musto P, Anderson KC, Attal M, Richardson PG, Badros A, Hou J (2018). Second primary malignancies in multiple myeloma: an overview and IMWG consensus. Ann Oncol.

[14] Ailawadhi S, Swaika A, Razavi P, Yang D, Chanan-Khan A (2014). Variable risk of second primary malignancy in multiple myeloma patients of different ethnic subgroups. Blood Cancer J.

[15] Fei F, Reddy V, Rosenblum F (2021). Secondary primary malignancies in patients with multiple myeloma: a single institution experience. Hematol Oncol.

