# Supplementary material for: Racial differences as predictors of outcomes in young patients with multiple myeloma
Source: Blood Cancer J. 2022 Jul 27;12(7):114. doi: 10.1038/s41408-022-00708-3 (PMC9329432; doi:10.1038/s41408-022-00708-3)
Supplement: Supplementary file 1 — Supplemental Material (tables and figures) [file 41408_2022_708_MOESM1_ESM.pdf]

Supplemental materials of
Racial differences as predictors of outcomes in young patients with multiple myeloma

Authors: Alicia Bao[1], Qiuhong Zhao[2], Elizabeth Merritt[3], Naresh Bumma[2], Srinivas Devarakonda[2], Abdullah M. Khan[2], Elvira Umyarova[2], Ashley E. Rosko[2], Don M. Benson[2], and Francesca Cottini[2*]

Affiliations:

[1] College of Medicine, The Ohio State University, Columbus, OH
[2] Department of Internal Medicine, Division of Hematology, College of Medicine, The Ohio State University, Columbus, OH
[3] The Ohio State University, Columbus, OH

To whom correspondence should be addressed*:

Francesca Cottini, MD

Assistant Professor of Medicine

Division of Hematology

The Ohio State University

College of Medicine

400 West 12th Avenue

Columbus OH 43210-1240 USA

Email: francesca.cottini@osumc.edu

phone: 614-292-6818

Supplemental Material Content:

Supplementary Table S1. Patient, disease, and treatment characteristics (n = 258).

Supplementary Table S2. Type and time of occurrence of second malignancy in non-Hispanic White and non-Hispanic Black patients with Multiple Myeloma (MM).

Supplementary Table S3. Progression-free survival (PFS) rates with 95% Confidence Intervals (CI) in non-Hispanic White and non-Hispanic Black patients at 1, 3, 5, and 10 years.

Supplementary Table S4. Overall survival (OS) rates with 95% Confidence Intervals (CI) in non-Hispanic White and non-Hispanic Black patients at 1, 3, 5, 10, 15, and 20 years.

Supplementary Figure S1. Outcomes of non-Hispanic White patients (NHWP) and non-Hispanic Black patients (NHBP) with MM diagnosed before age 50, stratified based on induction regimen.

Supplementary Figure S2. Overall survival (OS) outcomes of non-Hispanic White patients (NHWP) and non-Hispanic Black patients (NHBP) with MM diagnosed before age 50 without a second malignancy.

Supplementary Figure S3. Overall survival (OS) outcomes of non-Hispanic White patients (NHWP) and non-Hispanic Black patients (NHBP) with MM diagnosed before age 50, stratified based on the presence of del(13q).

Supplementary Table S1. Patient, disease, and treatment characteristics (n = 258).

	All (n = 258)	Non-Hispanic White patients (n = 198)	Non-Hispanic Black patients (n = 60)	*P-value*
**Age (median, range)**	46 (17-50)	46 (17-50)	45 (34-50)	0.32
< 30 years (no-%)	6 (2.3)	6 (3.0)	0 (0)	
30-40 years (no-%)	35 (13.6)	23 (11.6)	12 (20)	
40-50 years (no-%)	217 (84.1)	169 (85.4)	48 (80)	
**Gender (no-%)**				0.18
Male	165 (64.0)	131 (66.2)	34 (56.7)	
Female	93 (36.0)	67 (33.8)	26 (43.3)	
**Year of diagnosis (no-%)**				0.08
1992-2002	25 (9.6)	23 (11.6)	2 (3.3)	
2003-2019	233 (90.4)	175 (88.4)	58 (96.7)	
**ISS stage (no-%)**				0.11
Stage I	89 (42.0)	66 (40.0)	23 (48.9)	
Stage II	66 (31.1)	49 (29.7)	17 (36.2)	
Stage III	57 (26.9)	50 (30.3)	7 (14.9)	
NA	46	33	13	
**MM type (no-%)**				0.37
IgG	121 (46.9)	89 (44.9)	32 (53.3)	
IgA	53 (20.5)	44 (22.2)	9 (15.0)	
Light chain disease	72 (27.9)	56 (28.3)	16 (26.7)	
Other	12 (4.7)	9 (4.5)	3 (5.0)	
**Presentation (no-%)**				0.56
MM	201 (77.9)	151 (76.3)	50 (83.3)	
EMD-Bone	28 (10.9)	22 (11.1)	6 (10)	
EMD	21 (8.1)	17 (8.6)	4 (6.7)	
PCL	6 (2.3)	6 (3.0)	0 (0.0)	
Amyloidosis	2 (0.8)	2 (1.0)	0 (0.0)	
**Cytogenetics (no-%)**	**(n = 210)**	**(n = 159)**	**(n = 51)**	
t(11;14)	42 (20)	28 (17.6)	14 (27.5)	0.13
t(4;14)	15 (7.1)	13 (8.2)	2 (3.9)	0.30
t(8;14)	4 (1.9)	4 (2.5)	0 (0.0)	0.25
Del(17p)	15 (7.1)	13 (8.2)	2 (3.9)	0.30
Del(13q)	72 (34.3)	62 (39.0)	10 (19.6)	0.01
1q21+	48 (22.9)	37 (23.3)	11 (21.6)	0.80
Hypodiploidy	5 (2.4)	2 (1.3)	3 (5.9)	0.09
**Risk category (no-%)**				0.37
Standard (0 HRCA)	147 (70.0)	111 (68.9)	36 (73.5)	
High-risk (1 HRCA)	49 (23.3)	37 (23.0)	12 (24.5)	
Ultra-high risk (≥ 2 HRCA)	14 (6.7)	13 (8.1)	1 (2.0)	
**Induction therapy (no-%)**				0.94
Bortezomib-dex (VD)	57 (22.1)	44 (22.2)	13 (21.7)	
Lenalidomide-dex (RD)	25 (9.7)	19 (9.6)	6 (10.0)	
Bortezomib-lenalidomide-dex (VRD)	70 (27.1)	52 (26.3)	18 (30.0)	
Other	106 (41.1)	83 (41.9)	23 (38.3)	
**Transplant (no-%)**				0.11
No	20 (7.8)	13 (6.6)	7 (11.7)	
One ASCT	195 (75.6)	149 (75.3)	46 (76.7)	
Two or more ASCT	30 (11.6)	23 (11.3)	7 (11.7)	
ASCT followed by Allogeneic	13 (5.0)	13 (6.6)		
**Maintenance after ASCT (no-%)**	**(n = 238)**	**(n = 185)**	**(n = 53)**	0.31
No	120 (50.4)	90 (48.6)	24 (56.6)	
Yes	118 (49.6)	95 (51.4)	21 (43.4)	
**Lines of therapy (median, range)**	3 (1-21)	3 (1-15)	3 (1-21)	0.82
**Family History of cancer (no-%)**	**(n = 221)**	**(n = 172)**	**(n = 49)**	
Negative	99 (44.8)	71 (41.3)	28 (57.1)	0.05
Positive other than MM	115 (52.0)	95 (55.2)	20 (40.8)	0.10
Positive for MM/MGUS	7 (0.03)	6 (3.4)	1 (2.0)	0.99
**Personal history of cancer (no-%)**	**(n = 226)**	**(n = 176)**	**(n = 50)**	
No	203 (89.8)	154 (87.5)	49 (98.0)	0.03
Before MM diagnosis	10 (4.4)	9 (5.1)	1 (2.0)	0.70
After MM diagnosis	13 (5.8)	13 (7.4)	0 (0.0)	0.08
**Smoking (no-%)**	**(n = 251)**	**(n = 193)**	**(n = 58)**	0.37

No	144 (57.3)	114 (59.1)	30 (51.8)	
Yes (Active or previous at diagnosis)	107 (42.7)	79 (40.9)	28 (48.2)	

Abbreviations: ISS- International staging system, MM- multiple myeloma, EMD- extramedullary disease, PCL- plasma cell leukemia, HRCA- high-risk chromosomal abnormalities, dex- dexamethasone, ASCT- autologous stem cell transplant, MGUS- monoclonal gammopathy of undetermined significance.

Supplementary Table S2. Type and time of occurrence of second malignancy in non-Hispanic White and non-Hispanic Black patients with Multiple Myeloma (MM).

Second malignancy	n = 226	Non-Hispanic White patients (n = 176)	Non-Hispanic Black patients (n = 50)	*P-value*
**Before MM diagnosis (no-%)**	10 (4.4)	9 (5.1)	1 (2.0)	0.70
**Second malignancy type**		1) Hairy cell leukemia<br>2) Melanoma<br>3) Cervical cancer<br>4) Endometrial cancer<br>5) CLL<br>6) Papillary thyroid cancer<br>7) Squamous cell carcinoma of the skin<br>8) Mantle cell lymphoma<br>9) Meningioma	1) Cervical cancer	
**After MM diagnosis (no-%)**	13 (5.8)	13 (7.4)	0 (0)	0.08
**Second malignancy type**		1) Renal cell carcinoma<br>2) Colorectal cancer [2]<br>3) Cutaneous T-cell lymphoma<br>4) Myelodysplastic syndrome<br>5) Basal cell carcinoma [2]<br>6) B-ALL [2]<br>7) DLBCL<br>8) Schwannoma<br>9) Papillary thyroid cancer<br>10) Squamous cell carcinoma of the skin		

Abbreviations: CLL, chronic lymphocytic leukemia, B-ALL, B-cell acute lymphoblastic leukemia, DLBCL, diffuse large B-cell lymphoma.

Supplementary Table S3. Progression-free survival (PFS) rates with 95% Confidence Intervals (CI) in non-Hispanic White and non-Hispanic Black patients at 1, 3, 5, and 10 years.

Non-Hispanic White patients				Non-Hispanic Black patients			
Year	PFS rate from diagnosis	95% CI		Year	PFS rate from diagnosis	95% CI	
1	91%	86%	95%	1	98%	86%	95%
3	54%	47%	61%	3	65%	52%	76%
5	38%	31%	45%	5	50%	36%	62%
10	22%	16%	29%	10	26%	14%	39%
Median PFS	3.2	2.8	4.3	Median PFS	5.9	3.0	6.2
Year	PFS rate from first transplant	95% CI		Year	PFS rate from first transplant	95% CI	
1	74%	67%	79%	1	81%	67%	89%
3	45%	38%	52%	3	54%	39%	67%
5	33%	26%	40%	5	47%	33%	60%
10	18%	13%	25%	10	26%	14%	40%
Median PFS	2.4	1.8	3.5	Median PFS	3.3	2.0	5.8

Supplementary Table S4. Overall survival (OS) rates with 95% Confidence Intervals (CI) in non-Hispanic White and non-Hispanic Black patients at 1, 3, 5, 10, 15, and 20 years.

Non-Hispanic White				Non-Hispanic Black			
Year	OS rate from diagnosis	95% CI		Year	OS rate from diagnosis	95% CI	
1	99%	96%	100%	1	98%	89%	100%
3	82%	76%	87%	3	91%	80%	96%
5	66%	58%	72%	5	86%	73%	93%
10	47%	39%	54%	10	54%	37%	68%
15	30%	21%	39%	15	54%	37%	68%
20	14%	6%	24%	20	NR	NR	NR
Median OS	8.6	6.9	12.1	Median OS	NR	7.4	NR
Year	OS rate from first transplant	95% CI		Year	OS rate from first transplant	95% CI	
1	93%	88%	96%	1	92%	81%	97%
3	73%	66%	79%	3	84%	71%	92%
5	61%	53%	68%	5	75%	60%	85%
10	44%	36%	52%	10	52%	35%	66%
15	26%	17%	36%	15	52%	35%	66%
20	9%	2%	21%	20	NR	NR	NR
Median OS	7.3	5.5	11.4	Median OS	NR	6.1	NR

Abbreviations: NR, not reached

Figure S1.

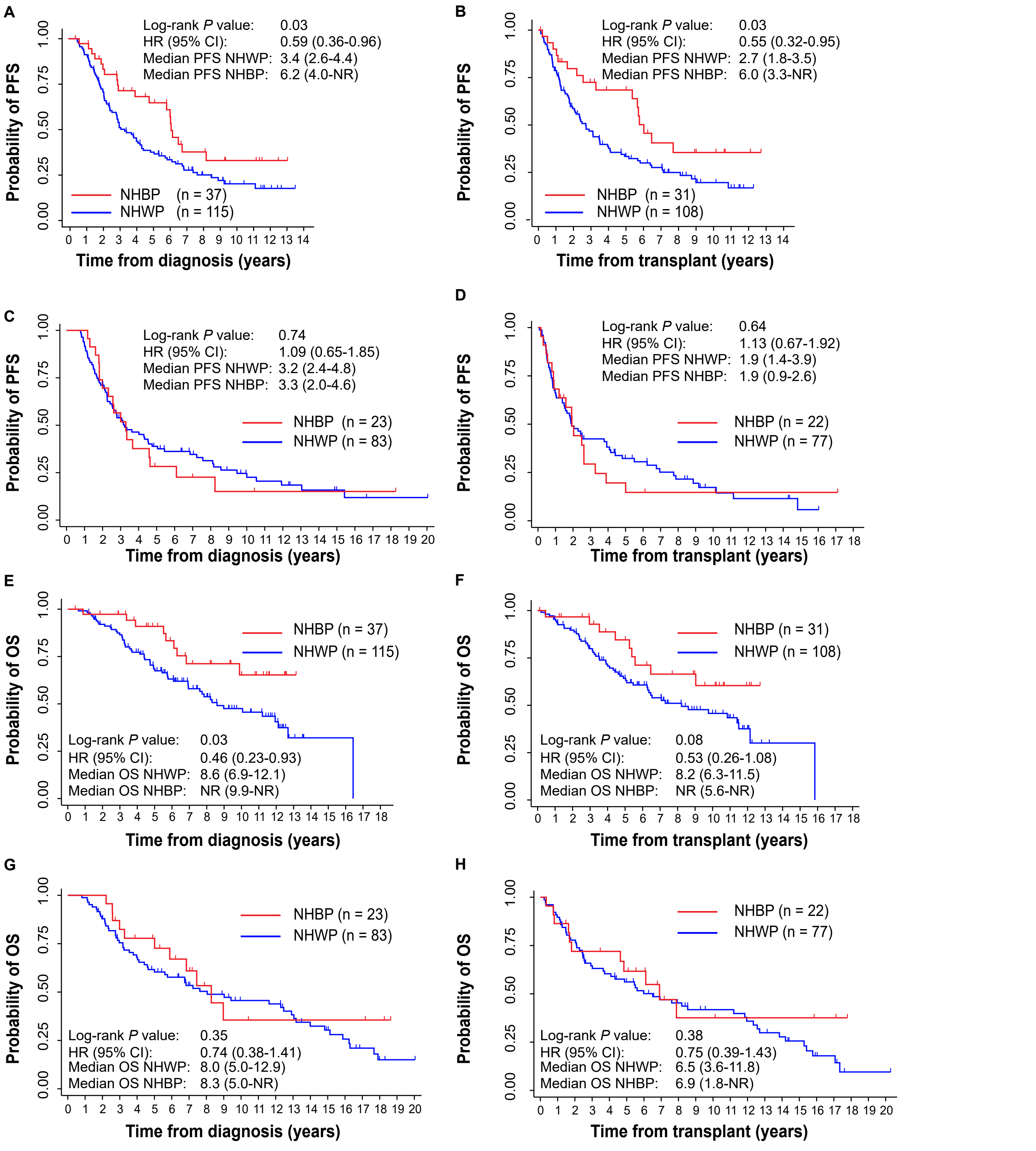

Figure S1. Outcomes of non-Hispanic White patients (NHWP) and non-Hispanic Black patients (NHBP) with MM diagnosed before age 50, stratified based on induction regimen.
Kaplan-Meier curves of progression-free survival (PFS) from diagnosis or first transplant in NHWP and NHBP who received VD-RD-VRD (**A-B**) or other therapies (**C-D**) as induction strategy;
Kaplan-Meier curves of overall survival (OS) from diagnosis or first transplant in NHWP and NHBP who received VD-RD-VRD (**E-F**) or other therapies (**G-H**) as induction strategy. V, bortezomib; R, lenalidomide; D, dexamethasone; *P*, log-rank p-value; HR, Hazard Ratio; CI, Confidence Interval; NR, not reached.

Figure S2.

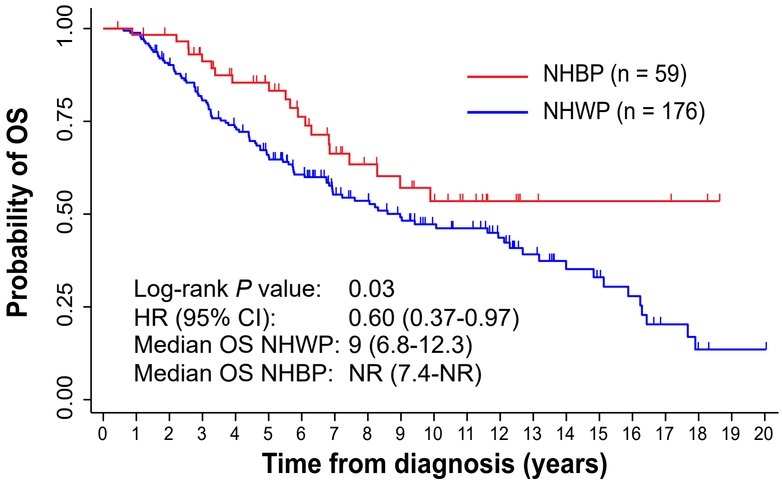

Figure S2. Overall survival (OS) outcomes of non-Hispanic White patients (NHWP) and non-Hispanic Black patients (NHBP) with MM diagnosed before age 50 without a second malignancy.
Kaplan-Meier curves of OS from diagnosis in n = 235 patients. *P*, log-rank p-value; HR, Hazard Ratio; CI, Confidence Interval; NR, not reached.

Figure S3.

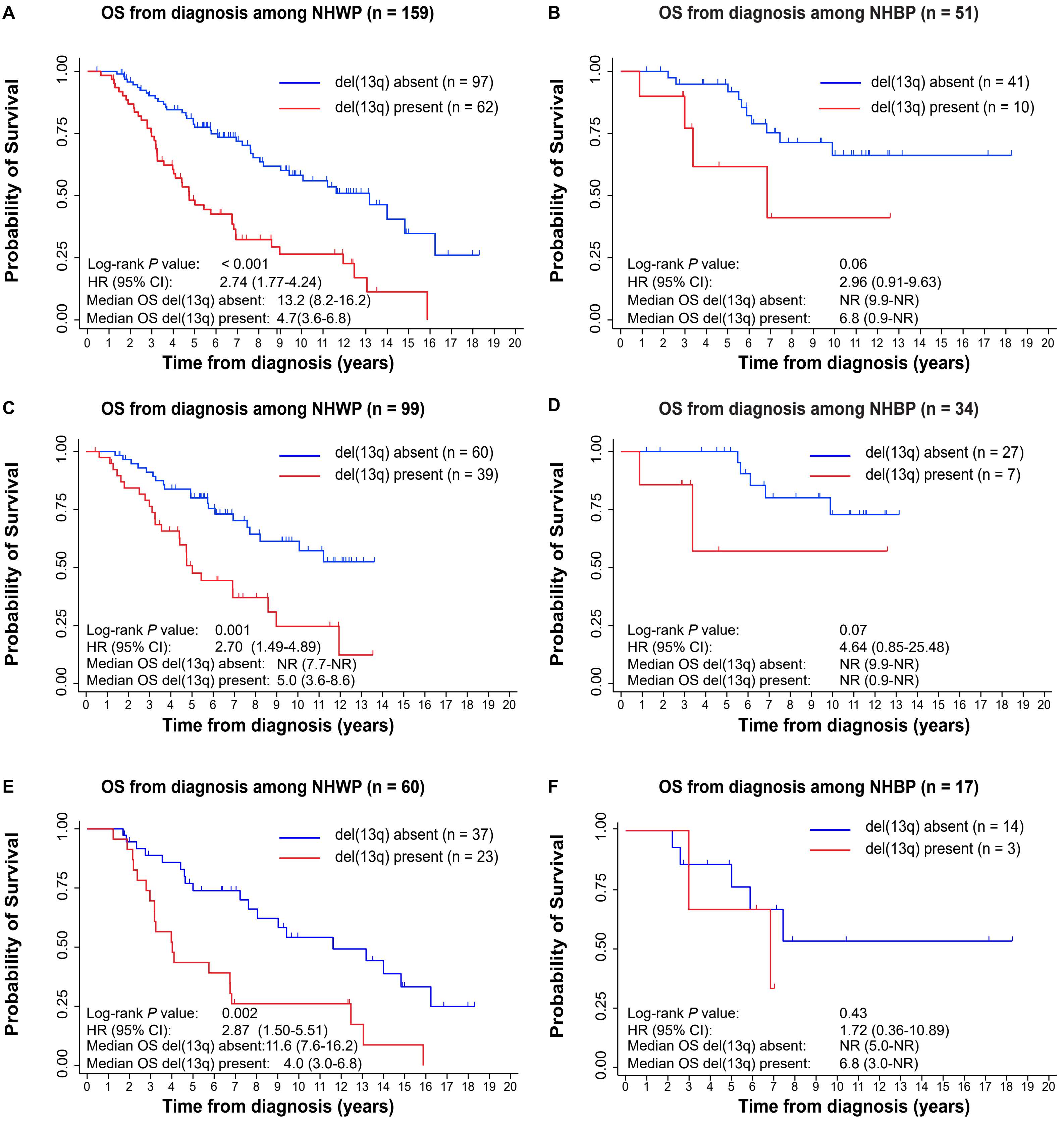

Figure S3. Overall survival (OS) outcomes of non-Hispanic White patients (NHWP) and non-Hispanic Black patients (NHBP) with MM diagnosed before age 50, stratified based on the presence of del(13q). Kaplan-Meier curves of OS from diagnosis based on the presence or absence of del(13q) in all NHWP (**A**) or NHBP (**B**), in NHWP (**C**) or NHBP (**D**) treated with VD-RD-VRD, and in NHWP (**E**) or NHBP (**F**) treated with other induction therapies. *P*, log-rank p-value; HR, Hazard Ratio; CI, Confidence Interval; NR, not reached.
